# Mild synthesis of ultra-bright carbon dots with solvatochromism for rapid lipid droplet monitoring in varied physiological processes

**DOI:** 10.1093/rb/rbad109

**Published:** 2024-01-16

**Authors:** Borui Su, Dong Gao, Nini Xin, Kai Wu, Mei Yang, Shichao Jiang, Yusheng Zhang, Jie Ding, Chengheng Wu, Jing Sun, Dan Wei, Hongsong Fan, Zhenzhen Guo

**Affiliations:** National Engineering Research Center for Biomaterials, College of Biomedical Engineering, Sichuan University, Chengdu, Sichuan 610064, China; National Engineering Research Center for Biomaterials, College of Biomedical Engineering, Sichuan University, Chengdu, Sichuan 610064, China; National Engineering Research Center for Biomaterials, College of Biomedical Engineering, Sichuan University, Chengdu, Sichuan 610064, China; National Engineering Research Center for Biomaterials, College of Biomedical Engineering, Sichuan University, Chengdu, Sichuan 610064, China; National Engineering Research Center for Biomaterials, College of Biomedical Engineering, Sichuan University, Chengdu, Sichuan 610064, China; National Engineering Research Center for Biomaterials, College of Biomedical Engineering, Sichuan University, Chengdu, Sichuan 610064, China; National Engineering Research Center for Biomaterials, College of Biomedical Engineering, Sichuan University, Chengdu, Sichuan 610064, China; National Engineering Research Center for Biomaterials, College of Biomedical Engineering, Sichuan University, Chengdu, Sichuan 610064, China; National Engineering Research Center for Biomaterials, College of Biomedical Engineering, Sichuan University, Chengdu, Sichuan 610064, China; Institute of Regulatory Science for Medical Devices, Sichuan University, Chengdu, Sichuan 610064, China; National Engineering Research Center for Biomaterials, College of Biomedical Engineering, Sichuan University, Chengdu, Sichuan 610064, China; National Engineering Research Center for Biomaterials, College of Biomedical Engineering, Sichuan University, Chengdu, Sichuan 610064, China; National Engineering Research Center for Biomaterials, College of Biomedical Engineering, Sichuan University, Chengdu, Sichuan 610064, China; Department of Gastroenterology, Sichuan Provincial People’s Hospital, University of Electronic Science and Technology of China, Chengdu 610072, China

**Keywords:** lipid droplet probe, carbon dots, fluorescence imaging, wash-free, dynamic monitoring

## Abstract

Lipid droplets (LDs) participating in various cellular activities and are increasingly being emphasized. Fluorescence imaging provides powerful tool for dynamic tracking of LDs, however, most current LDs probes remain inconsistent performance such as low Photoluminescence Quantum Yield (PLQY), poor photostability and tedious washing procedures. Herein, a novel yellow-emissive carbon dot (OT-CD) has been synthesized conveniently with high PLQY up to 90%. Besides, OT-CD exhibits remarkable amphiphilicity and solvatochromic property with lipid–water partition coefficient higher than 2, which is much higher than most LDs probes. These characters enable OT-CD high brightness, stable and wash-free LDs probing, and feasible for *in vivo* imaging. Then, detailed observation of LDs morphological and polarity variation dynamically in different cellular states were recorded, including ferroptosis and other diseases processes. Furthermore, fast whole imaging of zebrafish and identified LD enrichment in injured liver indicate its further feasibility for *in vivo* application. In contrast to the reported studies to date, this approach provides a versatile conventional synthesis system for high-performance LDs targeting probes, combing the advantages of easy and high-yield production, as well as robust brightness and stability for long-term imaging, facilitating investigations into organelle interactions and LD-associated diseases.

## Graphical Abstract

**Figure:**
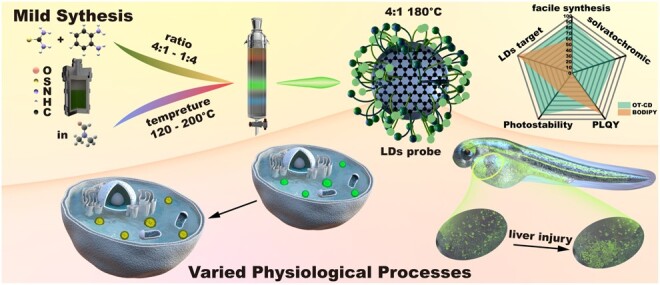

## Introduction

Lipid droplets (LDs) are organelles that originate from the endoplasmic reticulum and are composed of a hydrophobic core of neutral lipids and a phospholipid monolayer modified by various proteins [1]. They are present in almost all types of cells with distinguishable differences in composition [2]. LDs participate in regulating various cellular activities [3], and in addition to their involvement in lipid and protein metabolism, LDs sequester transcription factors or chromatin components play a role in viral assembly, or store antibody proteins in immunization processes [4]. It is notable that the morphology and quantity of LDs within cells undergo significant changes in individuals afflicted with metabolic syndrome, cancer, and neurodegenerative diseases [5–9]. Additionally, LDs have been shown to function as antioxidant organelles, mitigating oxidative stress and cell death by actively regulating the trafficking of polyunsaturated fatty acids, while also promoting ferroptosis through the process of autophagy (lipophagy) [10]. Taken together, LDs are involved in numerous cellular activities and exhibited morphological and compositional differences. Therefore, real-time monitoring of LDs dynamics is essential to gain new insights into pathological mechanisms [11–13].

Fluorescence imaging is a widely employed and efficacious method for observing live cells, providing high-resolution images while causing minimal cellular damage [14]. By utilizing diverse fluorescent probes, this technique can discriminate the object of interest from a complex background [15–18]. Compared to fluorescent small molecules prone to photoquenching and quantum dots containing heavy metal elements, carbon dots (CDs) are considered excellent candidates for the development of fluorescent probes because of their large stokes shift, high resistance to photobleaching, high Photoluminescence Quantum Yield (PLQY), and low biotoxicity [19–26]. Solvothermal method is a prevailing approach for synthesizing CDs. Initially, the precursor small molecules undergo molecular aggregation, resulting in the formation of larger molecules or sparsely distributed nanoscale polymers. Subsequently, through progressive dehydration and carbonization with extended reaction time, a carbonized core with a size on the order of a few nanometers is ultimately formed [27]. The surface of the carbonized core, retaining common functional groups, precursor or intermediate molecules, etc., typically exhibits sparser structures and defects [28]. Thus, through the rational design of reaction system, CDs with desired properties can be customized [29]. For instance, by summarizing the property variations of all products under diverse conditions, it was observed that higher temperature, increased pressure and prolonged reaction time promote the generation of larger carbonized core with red-shifted fluorescence emission [30, 31]. In addition, the surface state structure of carbonized core allows them to be multifunctional designed and easily tuned into certain properties. For example, introducing various elements as dopants into the carbonized core can produce different structures or defects, and adjusting the reaction conditions can affect the doping ratio to a certain extent [32]. Similarly, the use of different precursor reactions can lead to different surface states on the carbonized core. Also, modifying the reaction conditions can change the composition ratios of these surface states and the resulting contact patterns [33–36]. These regulatory ways provide an interesting insight to design CDs with different properties. Based on that CD properties are extensive tunable, we try to explore the solvothermal reaction process by adjusting the precursor ratio as well as the reaction temperature, aiming to seek the synthesis of new CDs with high performance under milder condition. Then we get a new yellow emitted CD, noted as OT-CD, which could be obtained below 200°C, whereas exhibit excellent amphiphilicity and solvatochromic properties, thus possible for fast and longtime LDs targeting [37, 38].

**Scheme 1.**
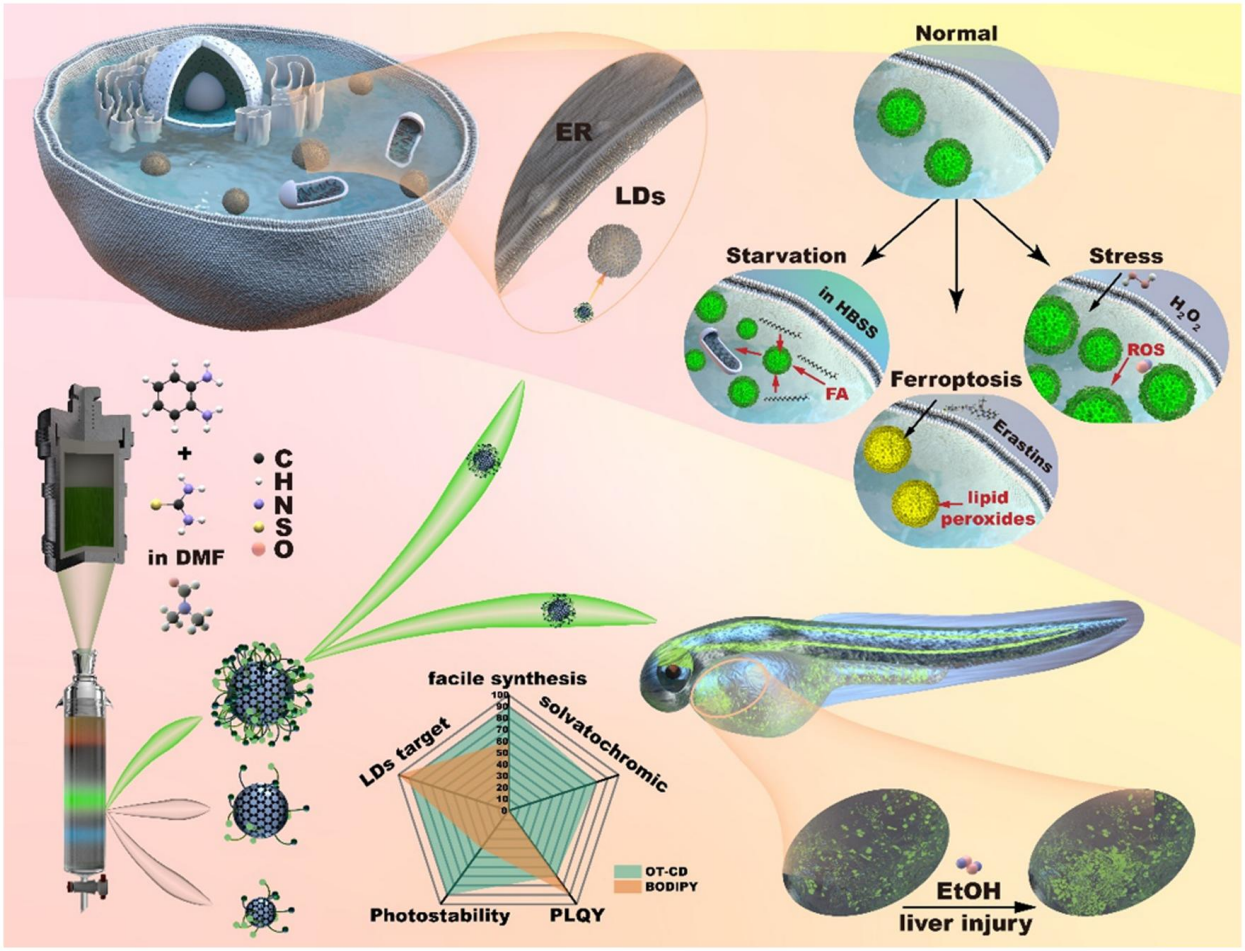
Screening of OT-CD for rapid LDs imaging in varied physiological processes.

To gain deep insights into the relationship between the formation process of OT-CD and the reaction conditions, we first tried to prepare varied OT-CDs under three precursor ratios and five reaction temperatures following by column chromatography separating. Then by comparing the excitation emission matrices of OT-CD obtained under different conditions, we explored the emission mechanism of OT-CD and the effect of reaction conditions on both the relative yield and the content of molecular states in OT-CD, as well as the significant differences in fluorescence properties. The water and lipid solubility, lipidwater partition coefficient and solvatochromic effect were also explored. Finally, we investigated the fast and accurate LDs targeting ability of OT-CD by imaging different types of cells and zebrafish, as well as the stability for dynamic observation. To confirm the possibility of this OT-CD for real-time monitoring of LDs dynamics in pathological process, we observed the variation of LDs polarity during Erastin-induced iron toxicity in Hela cells by tracking the fluorescence changes of OT-CD at different wavelengths (Scheme 1). This study not only inspires the development of synthetic routes for CDs, but also provides a valuable tool for understanding LD-related diseases process effectively.

## Experimental section

### Chemicals and material

O-phenylenediamine (oPD), thiourea, glyceryl trioleate and Erastin were obtained from Aladdin Reagent Co. n-Octano and oleic acid were obtained from macklin, ShangHai. Dioxane, dichloromethane (DCM), acetone, N, N-dimethylformamide (DMF) and dimethyl sulfoxide (DMSO), ethanol and methanol were purchased from Kelong Chemical Reagent Factory Chengdu, Quinine. Sulfate was obtained from Bide Pharmatech Co., Ltd. BODIPY493/503 was obtained from Thermo Fisher. RedDotTM1 came from Biotium. Mito Tracker Red, Lyso-Tracker Red and ER-Tracker Red were purchased from Beyotime Institute of Biotechnology, Shanghai, China. Dulbecco's Modified Eagle Medium (DMEM), alpha-Modified Eagle Medium and Hank’s balanced salt solution (HBSS) media came from Hyclone (USA). MTT (3-(4,5-dimethylthiazol-2-yl)-2,5-diphenyltetrazoli-um bromide) was purchased from Beijing Solarbio Science and Technology, China. All chemicals were analytical grade and used without any further purification.

### Synthesis of OT-CDs

OT-CDs were synthesized via a one-pot solvothermal treatment using various ratios of o-PDs and thiourea, with a total molar amount of 3 mmol in 30 ml DMF. Subsequently, the mixed solution was transferred to a 100 ml polytetrafluoroethylene (Teflon)-lined autoclave and heated at five temperatures (120, 140, 160, 180 and 200°C) for 8 h, respectively After cooling down to room temperature, the reacted solution was filtered with 0.22 μm micropore membranes to remove large particles. Next, the obtained solution was concentrated, followed by a column chromatography using dichloromethane as the eluent to purify the product. Finally, the yellow-emitting component was collected and green powders were obtained after lyophilization. All OT-CDs show the same bright yellow fluorescence in the silica gel column under 365 nm UV light, and emitted a brighter green fluorescence in dichloromethane after the collection is completed. (Supplementary Figure S1) All collected OT-CDs were spin evaporated and then re-dissolved in ethanol and stored away from light.

### Cell culture and cytotoxicity study

HeLa cells (Cervical Cancer cells), HepG2 cells (Hepatocellular Carcinoma cells), MG63 cells (Human Bone Osteosarcoma cell) and MC3T3 cells (Mouse osteoblastic cell) were cultured in DMEM supplemented with 10% fetal bovine serum and 1% penicillin and streptomycin. All above cells were cultured in an incubator with the environmental conditions of 5% CO_2_ at 37°C. Before cell staining, all cells were plated on glass bottom cell culture dish and allowed to adhere for 24 h.

The cytotoxicity of OT-CD against Hela cells was assessed by standard MTT assay. In brief, the cells were cultured in 96-well plates with a density of 1 × 10^4^ cells per well overnight. Then, DMEM was removed and fresh DMEM containing different doses of OT-CD (0, 10, 20, 30, 40, 50 μg ml^−1^, respectively) was added. After 24 h, the medium was discarded. The cells were treated with 5 mg ml^−1^ MTT (20 μl well^−1^) followed by incubating for an additional 4 h at 37°C. Next, DMSO (200 μl well^−1^) was added to dissolve purple formazan salts. Finally, the absorbance was recorded using a multidetection microplate reader (Bio-Teklnstru-ments Inc., USA).

### Cell imaging and colocalization

The potential for cell imaging of the obtained OT-CDs was tested with HeLa cells, HepG2 cells, MG63 cells and MC3T3 cells, respectively. The cells (1 × 10^5^ cells ml^−1^) were seeded into a glass-bottomed cell culture dish and incubated for 24 h. In the colocalization experiment, BODIPY 493/503 (MTDR, 50 nM) was added and incubated for 30 min at 37°C for 30 min. Then, the cells were washed with phosphate-buffered saline three times to remove unbound dye molecules. After that, the cells were stained with OT-CD (10 μg ml^−1^) and directly used for imaging. The images were obtained using a Zeiss LSM 880 confocal laser scanning microscope (CLSM). OT-CD: λex = 405 nm. BODIPY 493/503: λex = 488 nm. The overlap coefficient was calculated using ZEN blue software.

### Monitoring polarity changes in LDs during ferroptosis

Hela cells (1 × 10^5^ cells ml^−1^) were first stained with 10 μM OT-CD for 30 min and then treated with erastin (10 μM) for 30 min to induce ferroptosis. Fluorescence imaging was performed with CLSM, and six emission channels were recorded at λex = 405 nm (λem_1_ = 480 ± 10 nm; λem_2_ = 500 ± 10 nm; λem_3_ = 520 ± 10 nm; λem_4_ = 540 ± 10 nm; λem_5_ = 560 ± 10 nm; λem_6_ = 580 ± 10 nm). The ratio of fluorescence was calculated using (λem_1_ + λem_2_ + λem_3_)/(λem_4_ + λem_5_ + λem_6_).

### Zebrafish imaging

Zebrafish embryos were purchased from Shanghai FishBio Co., Ltd. Zebrafish embryos at 6 hpf (hours post fertilization) were placed in culture dishes and incubated in deionized water enriched with methylene blue at 28°C. For CLSM imaging, zebrafish embryos at 6 hpf were stained with OT-CD and observed immediately. Additionally, zebrafish at four developmental stages (6, 12, 24 and 48 hpf) were stained with OT-CD for 20 min, and their images were recorded separately. The alcoholic liver injury model of zebrafish was established according to previous literature [39, 40]. Briefly, 96 hpf zebrafish larvae were transferred to an aqueous environment containing 2% ethanol and raised for 32 h. Zebrafish larvae were first anesthetized with 0.08% tricaine methanesulfonic acid solution, then fixed with 1% agarose gel. The experimental protocols were performed in accordance with the ARRIVE (Animal Research: Reporting of In Vivo Experiments) guidelines, and all animal experiments were approved by the Institutional Animal Care and Use Committee of Sichuan University.

## Result and discussion

### Formation process of OT-CDs

In our previous experiments, oPD and thiourea were dissolved in DMF, heated and subsequently separated by thin-layer chromatography (TLC). This process led to the discovery of a low-polarity OT-CD that exhibited remarkably bright fluorescence under 365 nm UV irradiation. Recognizing its potential for an extremely high photoluminescence quantum yield (PLQY) and LD targeting ability, we aimed to optimize the synthesis conditions to enhance the yield and overall performance of OT-CD.

**Figure 1.**
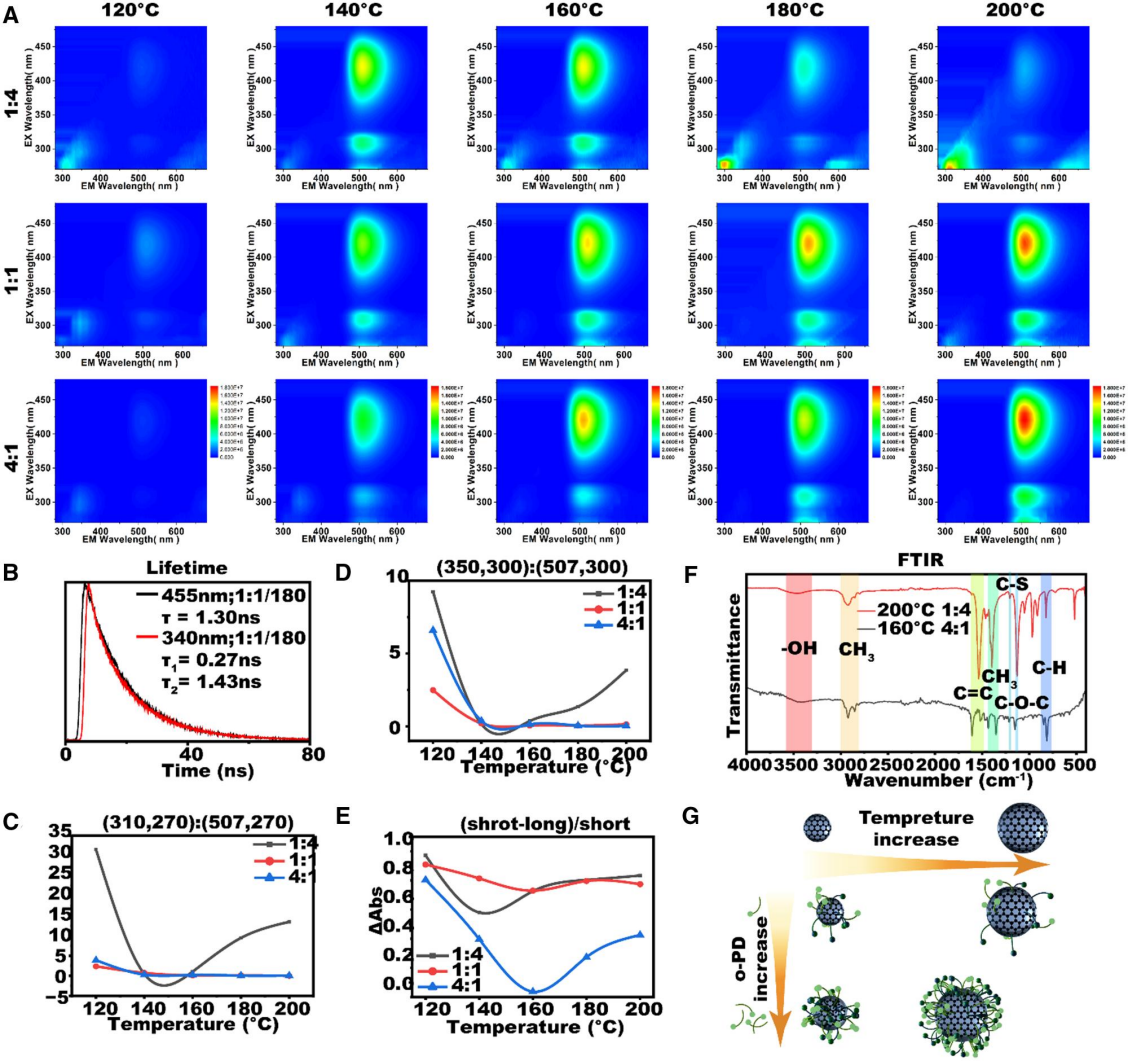
(**A**) EEM of all OT-CDs. (**B**) Fluorescence lifetime curves of OT-CDs under 340 and 455 nm excitation. (**C**) The ratio of EM intensity at 310 nm to that at 507 nm under 270 nm excitation. (**D**) The ratio of EM intensity at 350 nm to that at 507 nm under 300 nm excitation. (**E**) Ratio of long wavelength to short wavelength absorption for different OT-CDs. (**F**) FTIR comparison of two OT-CDs. (**G**) Schematic of the differences of OT-CDs synthesized at different temperature to precursor ratios.

The excitation-emission matrices (EEM) of the OT-CDs are presented in Figure 1A. Each image contains two types of fluorescence emission, one excitation-dependent at short wavelengths and the other excitation-independent at long wavelengths, generated by the carbonized cores and surface fluorescence states, respectively. This hypothesis is supported by fluorescence lifetime data presented in Figure 1B, which shows that the fluorescence decay curves of OT-CDs emitted at 506 nm under 340 and 455 nm laser irradiation can be fitted by single and double exponential, respectively. This indicates that for fluorescence emission at 506 nm, only one fluorescent center is involved in long-wavelength excitation, while two fluorescent centers are involved in short-wavelength excitation. Furthermore, the fluorescence lifetimes were nearly same for the OT-CDs synthesized under different conditions, regardless of excitation by short or long wavelengths (Supplementary Figure S2). Notably, OT-CDs obtained under diverse synthetic conditions exhibit virtual indistinguishability in both TLC and fluorescence lifetime curves. This observation leads us to believe that their basic structures may be highly similar.

For all three precursor ratios, when synthesized at 120°C, the fluorescence emission of all OT-CDs was weak, whereas the long-wavelength emission increased when raised to 140°C (Figure 1A). This phenomenon may be due to that the generating of the required fluorophores to compose OT-CD at lower temperatures is challenging, while the creation of other component CDs or molecules is dominated. When the reaction temperature exceeded 160°C (over the boiling point of 153°C of DMF), the pressure in the reactor increased rapidly and the fluorescence properties of OT-CDs obtained from the three precursor ratios exhibited different changes. As shown in Figure 1C and D, for OT-CDs with a precursor ratio of 1:4 (black line), the short-wave fluorescence emission corresponding to the carbonized core [41] gradually dominated with an increase in reaction temperature (2–15 times larger). For OT-CDs with a precursor ratio of 1:1 and 4:1 (red and blue lines), the long-wave fluorescence emission corresponding to the surface fluorophore always dominates. Remarkably, if the OT-CD (4:1/180°C) with predominantly fluorescent emission is purified and then continued to be heated at 180°C for 8 h, a new component of very polar blue-white emission is obtained, showing excitation-dependent emission from the carbon-nucleated state (Supplementary Figure S3). Considering that a tighter carbonized core is usually beneficial for improving the photostability [42, 43], we tested CDs synthesized at three temperatures and found that their fluorescence bursts diminished with increasing temperature (Supplementary Figure S4B).

We further measured the UV absorption curves of the OT-CDs generated under different conditions, which corroborated the EEM results, showing three major absorption peaks corresponding to π–π* and n–π* transitions of the conjugated structure, as well as surface state absorption [44] (Supplementary Figure S5). Since the absorbance near 507 nm is lower than that near 270 nm, the smaller the relative difference between the two indicates a higher relative abundance of surface fluorophores. According to Figure 1E, the smallest difference presented at the 4:1/160°C sample, indicating that it had the most abundant surface fluorophores. This conclusion was also supported by the FTIR data (Figure 1F), where OT-CD obtained at 200°C with a 1:4 precursor ratio showed much stronger c=c bond and C–O–C bond signals than OT-CD with a 4:1 ratio at 160°C, suggesting a more carbonated structure.

Based on the aforementioned results, we propose the following formation pattern of OT-CD obtained under different conditions (Figure 1G): the formation of both carbonized core and fluorophores on the nuclei requires o-PD and a small amount of thiourea. At low temperatures, the formation of carbonized core and fluorophores is sluggish, resulting in no significant differences among OT-CDs obtained with different precursor ratios. As the temperature increases to approximately 140°C, a substantial quantity of associated fluorophores can be generated, causing the gradually dominated fluorescence emission at 507 nm. Further increasing the temperature to the boiling point of DMF results in a gradual increase in carbonized core production rate as pressure inside the reactor increases. Insufficient o-PD supply results in a reduction in the number of generated fluorophores, finally leading to a gradual domination of short-wavelength emissions associated with carbonized core.

As mentioned above, under the synthesis conditions of 4:1/160°C and 4:1/180°C, the maximum conversion of the precursor to OT-CD-related fluorophores was realized (Supplementary Figure S4A). Nevertheless, the lack of carbonation at 160°C led to the production of OT-CDs with inadequate light stability (Supplementary Figure S4B). Therefore, considering the yield, photostability and safety, we optimized the synthesis of OT-CD under the relatively mild condition of 4:1/180°C. Hereinafter, unless stated otherwise, OT-CD denotes the yielded products of the yellow-green, low-polarity carbon-dot fraction at 4:1/180°C.

**Figure 2.**
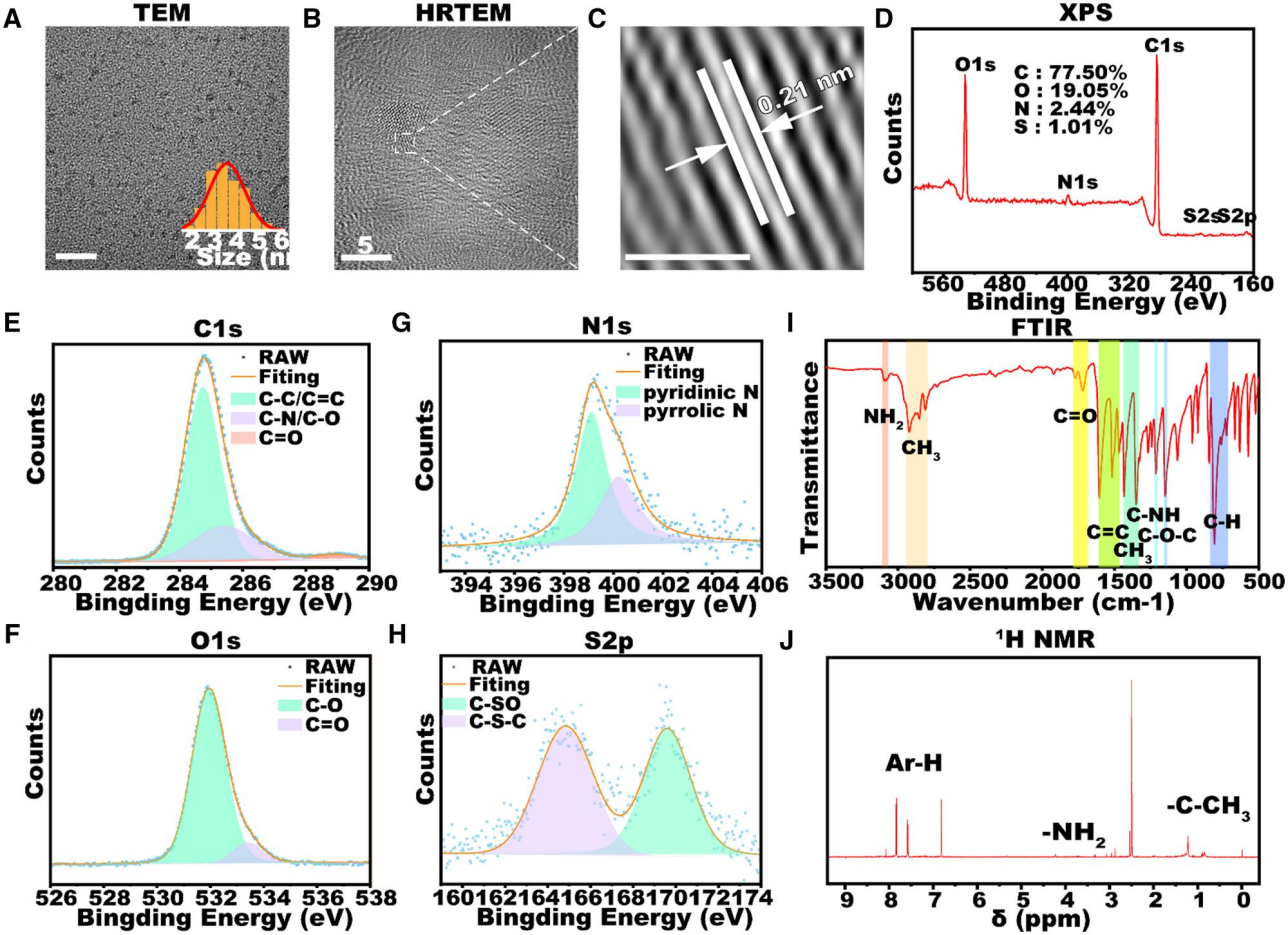
Structural characterization of OT-CD. (**A**) TEM images of OT-CD, and particle size distribution. (**B**) HRTEM image of OT-CD. (**C**) Enlarged and FFT transformed HRTEM image highlighting the 0.21 nm crystal plane spacing. (**D**) XPS spectra and high-resolution. (**E**) C 1 s, (**F**) O 1 s, (**G**) N 1 s and (**H**) S2p of OT-CD. (**I**) FTIR of OT-CD. (**J**) ^1^H NMR spectrum of OT-CD recorded in (CD3)2S=O.

TEM images show that OT-CD synthesized at 4:1/180°C exhibited a uniform spherical structure with an average particle size of 3.96 nm (Figure 2A). A clear 0.21 nm crystal plane spacing can be observed under high-resolution transmission electron microscopy (HRTEM) (Figure 2B and C), corresponding to the (100) crystal plane of graphite [45]. XPS analysis was further used to analyze the chemical composition of OT-CD. The full XPS spectrum (Figure 2D) shows that the OT-CD contains C, O, N and S elements, and the contents are 77.70, 19.05, 2.44 and 1.01%, respectively. The high-resolution spectrum confirms that three fitting peaks at 284.8, 285.4, and 283.9 eV from the C 1 s spectrum express the chemical structure of C–C/C=C, C–N/C–O and C=O (Figure 2E). Two fitting peaks at 531.9 and 533.4 eV from the O 1 s spectrum describe the existence of C=O and C–O bands, respectively (Figure 2F). Two fitting peaks from the N 1 s spectrum at 399.1 and 400.2 eV represent pyridinic N and pyrrolic N (Figure 2G). The S2p band can be deconvoluted into two peaks at 169.6 eV for C–SO and 164.75 eV for S–H [46] (Figure 2H). The above results together confirmed the element component and surface functional groups, hydroxyl, amino groups and ammonium cations on the surface of OT-CD.

The FTIR spectrum illustrates the presence of amino and methyl surface functional groups in OT-CD. As shown in Figure 2I, the –NH_2_ group gives rise to the stretching absorption band between 3045 and 3100 cm^−1^ [47]. The overlapping stretching absorption bands observed at 2920, 2850 and 2890 cm^−1^ correspond to surface-rich C–H groups [48], while the absorption peaks at 1430 and 1348 cm^−1^ correspond to the bending vibrations of C–H. The presence of these functional groups imparts good amphiphilicity to OT-CD, indicating possible LDs-targeting ability. The characteristic absorption peaks observed near 1600, 1511 and 1463 cm^−1^ mainly correspond to the C=C backbone of the benzene ring, while those near 680–860 cm^−1^ correspond to the out-of-plane bending vibrations of C–H on the benzene ring. Additionally, the absorption peaks observed at 1716, 1211 and 1145 cm^−1^ correspond to the stretching vibrations of C=O, C–NH, and C–O, respectively.

^1^H NMR spectrum was subsequently employed to verify the structure of OT-CD. As shown in Figure 2J, the signal in the range of 7–8 ppm should come from the H in the aromatic structure, while a weaker –NH_2_ peak appears at 4.32 ppm, indicating that the precursor group is partially retained, and the peak near 0.7–2.0 ppm corresponds to the H in the alkyl group.

In summary, the copious alkyl and amino groups present on the surface of OT-CD confer excellent amphiphilicity to the material. Furthermore, the extensive conjugated structure not only facilitates good solvatochromism, but also potentially enhances the overall stability of the material. Consequently, OT-CD is anticipated to serve as an outstanding polarity-responsive fluorescent probe.

### Fluorescence properties of OT-CD

**Figure 3.**
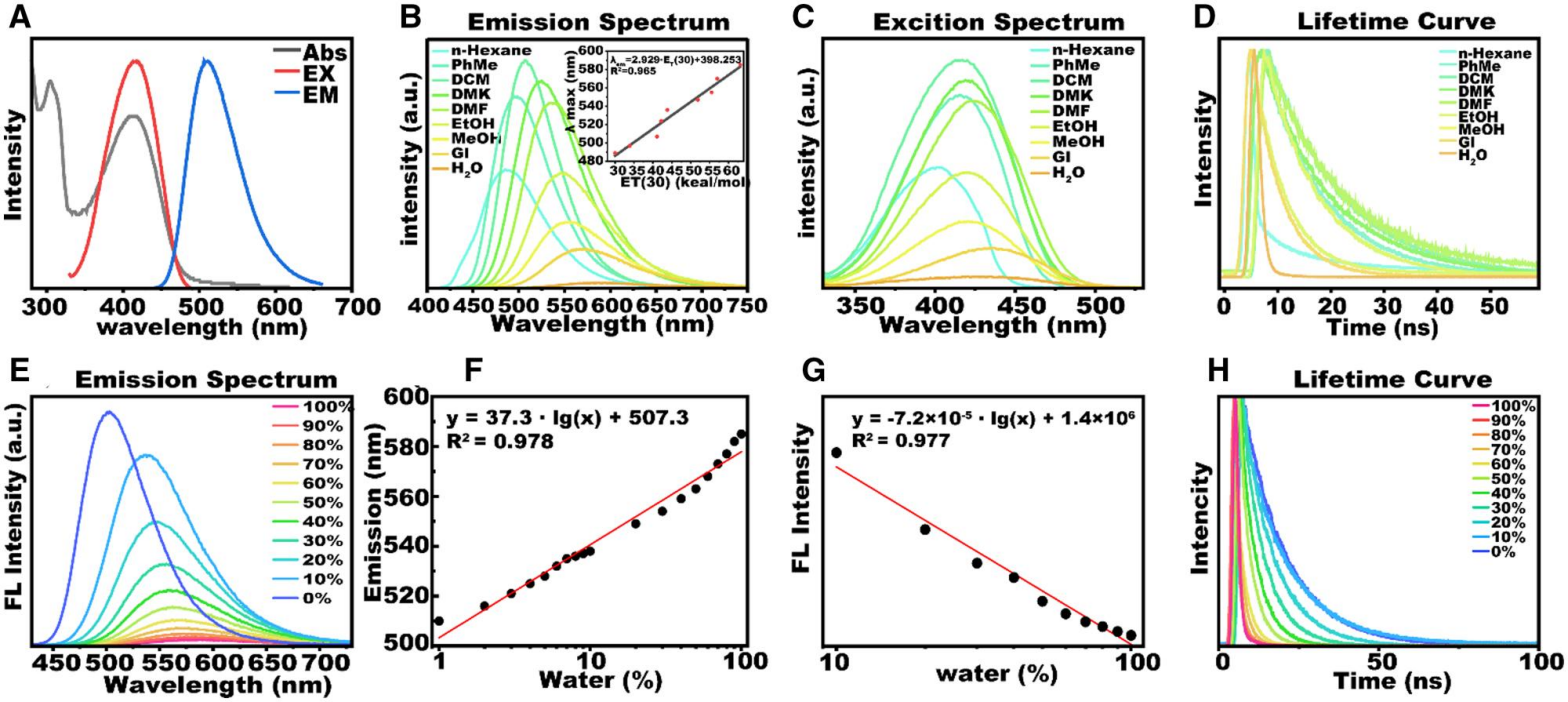
(**A**) Absorption, excitation and emission spectra of OT-CD. (**B**) Emission spectra, (**C**) excitation spectra and (**D**) fluorescence lifetime spectra of OT-CD in various solvents. (**E**) Emission spectra. Relationship between water content in 1,4-dioxane and maximum emission wavelength (**F**) as well as maximum fluorescence intensity (**G**). (**H**) Fluorescence lifetime spectra in the mixture of 1,4-dioxane and H_2_O.

The optical characteristics of OT-CD were subsequently investigated. The UV–Vis absorption spectra, excitation and emission spectra of OT-CD in dichloromethane are shown in Figure 3A. Consistent with the previous description, OT-CD exhibits mainly 507 nm emission excited by 420 nm light with a Stokes shift of about 90 nm. OT-CD also shows a significant solvatochromic effect, with their major emission peak red-shift from 489 to 585 nm as the solvent switched from cyclohexane to water (Figure 3B), and there is a good linear relationship between the emission peak position and the solvent polarity empirical constant [49]. The photograph of OT-CD dispersed in each solvent under sunlight and UV irradiation is shown in Supplementary Figure S6A. The fluorescence shows a clear change from blue to red with increasing polarity (Supplementary Figure S6B) and mostly has a high PLQY (Supplementary Figure S6C–K). Supplementary Table S1 lists some of the fluorescence property parameters of OT-CD in each solvent. The PLQY test result of 55.8% (Supplementary Figure S6L) for the standard sample of quinine sulfate is very close to the theoretical value of 54.6% [50], indicating a reliable test result. Meanwhile, the excitation peak position of OT-CD changes irregularly (Figure 3C). In addition, we tested the fluorescence lifetimes of OT-CD in different solvents under irradiation with a 455 nm laser (Figure 3D). Consistent with the variation of fluorescence emission intensity with solvent, the fluorescence lifetime showed an increasing trend followed by a decreasing trend (Supplementary Table S1).

To further quantify the polarity sensitivity of OT-CD, we measured their fluorescence properties in an H_2_O—1,4 dioxane hybrid system and fine-tuned the polarity of the solvent by varying the volume share of H_2_O in the hybrid system. As shown in Figure 3E–G, the emission wavelength of OT-CD red-shifted from λ = 502 nm in 1,4-dioxane to λ = 585 nm in H_2_O, and the fluorescence intensity also underwent a 97.8% decrease, and both change processes could be well fitted. Thus, we can easily distinguish solvents with different polarities based on the fluorescence emission of OT-CD or recording the change of polarity in the environment. In addition, the fluorescence lifetime of OT-CD was also found to be closely correlated with the change in polarity, with considerable potential for fluorescence lifetime imaging (Figure 3H).

**Figure 4.**
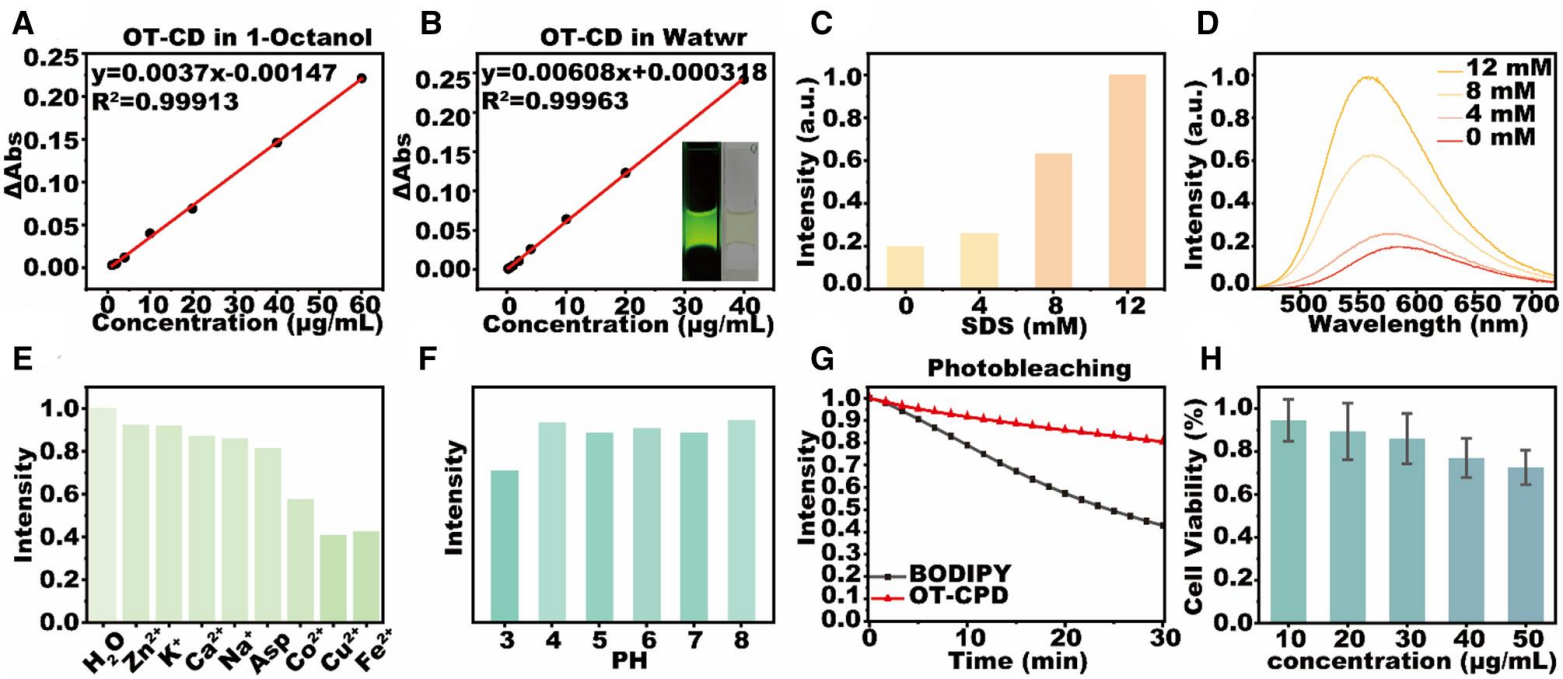
Absorption intensity-concentration curves of OT-CDs in n-octanol (**A**) and water (**B**). Fluorescence emission intensity (**C**) and fluorescence emission curves (**D**) of OT-CDs in aqueous sodium dodecyl sulfate solution at different concentrations. (**E**) Effect of different impurities in aqueous solution on the fluorescence emission intensity of OT-CDs. (**F**) Fluorescence emission intensity of OT-CDs in aqueous solutions with different pH values. (**G**) Fluorescence intensity profiles of OT-CDs and BODIPY with irradiation time. (**H**) Cytotoxicity test of different concentrations of OT-CD.

Based on the excellent fluorescence performance of OT-CD, we decided to further evaluate its potential as a fluorescent probe for biological application. Considering that OT-CD has a significant solvatochromic effect, we prospect that if OT-CD can also have some amphiphilicity, then it can present comparable contrast between water and oil in living organisms and obtain clear adipose imaging. We therefore tested that the lipid–water partition coefficient of OT-CD was 2.01, which is significantly larger than that of the commercial LDs probe BODIPY at 1.8 [51] (Figure 4A and B). It means that OT-CD not only possesses good amphipathic properties, but also tends to accumulate in lipid-like environments. Subsequently, we simulated LDs with various concentrations of SDS and observed a nearly 5-fold increase in fluorescence intensity after reaching the critical micelle concentration (Figure 4C and D), indicating that the solubilization effect of OT-CD can provide clear contrast in water–oil mixed systems.

We subsequently assessed the stability of OT-CD as a fluorescent probe and determined that common ions or amino acids had no significant effect on its fluorescence intensity, except for copper and iron ions (Figure 4E). Moreover, OT-CD exhibited consistent emission in an environment with a pH range of 4-8 (Figure 4F). These results suggest that OT-CD can maintain stable fluorescence performance in the complex and dynamic environments found in organisms. Interestingly, we tested the photobleaching resistance of commercial probes BODIPY and OT-CD in an aqueous environment and observed that the structure of OT-CD was significantly more photostable than that of BODIPY (Figure 4G).

Finally, we incubated Hela cells in different concentrations of OT-CD for 24 h and subjected them to MTT staining to assess cytotoxicity. We found that OT-CD concentration up to 30 μg ml^−1^ still possessed nearly 80% cell viability (Figure 4H), indicating the low cytotoxicity of our designed OT-CD, which is suitable for subsequent biofluorescence imaging experiments.

### Cell imaging and LDs monitoring

Based on the good optical properties and biocompatibility of OT-CDs, we further investigated its imaging ability in live cells by CLSM. First, HeLa cells were stained with OT-CD with different concentrations (10–50 μg ml^−1^). The CLSM images in Supplementary Figure S7 show the presence of many bright dot-like fluorescent signals in the cytoplasm under 405 nm laser excitation and homogeneous background signals in the rest. Notably, 10 μg ml^−1^ OT-CD could provide adequate imaging contrast, and this concentration is quite safe for the cells according to above MTT data. Hence, the following cell staining experiments were proceeded with 10 μg ml^−1^ of OT-CD.

**Figure 5.**
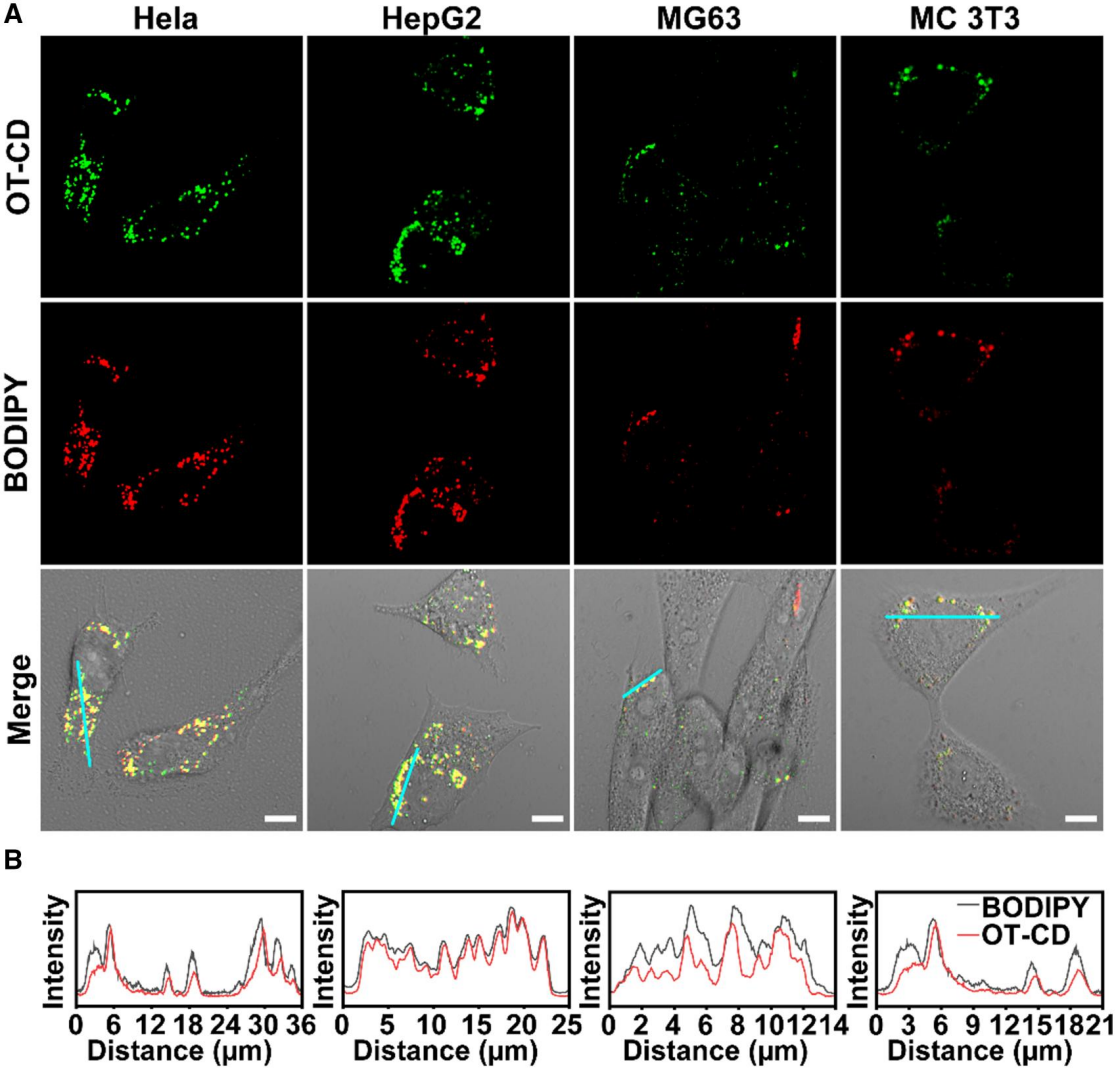
Fluorescence confocal images (**A**) and fluorescence intensity curves at different positions (**B**) of HeLa, HepG2, MG63 and MC3T3 after co-staining with OT-CD and BODIPY and merged bright field images. Scale bar: 10 μm.

To verify the LDs-specific target imaging ability of OT-CD, we performed co-localization experiments with the commercial probe BODIPY493/503 on four different sources of cells, Hela, HepG2, MG63 and MCT3T. As shown in Figure 5A and B and Supplementary Figure S8, the green fluorescence of OT-CD was consistent with that of BODIPY with an overlap factor of 0.9, confirming that OT-CDs can target intracellular LDs well in various cells.

**Figure 6.**
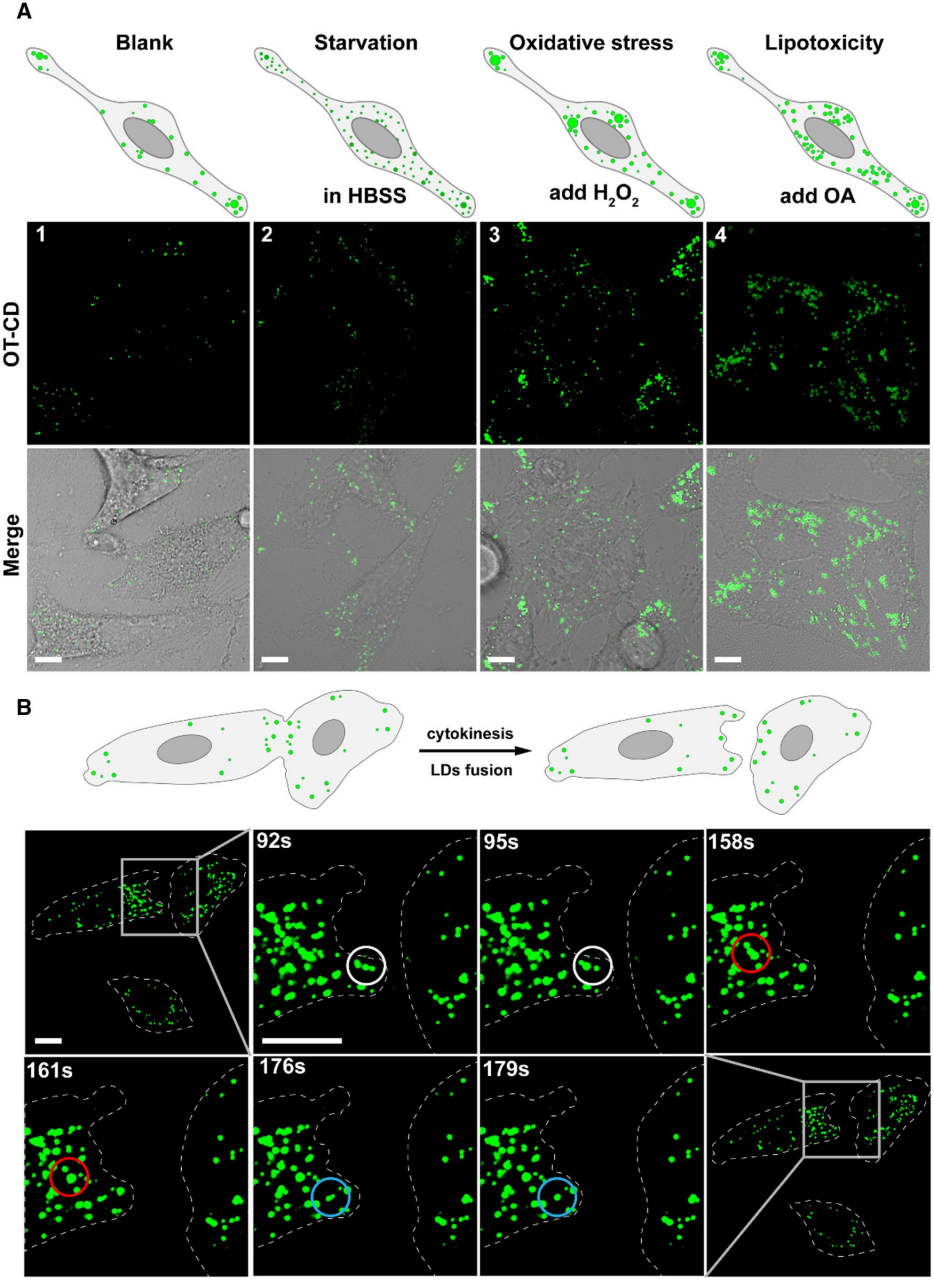
(**A**) FLSM images of HeLa cells under normal condition (1), starvation (2), oxidative stress 3) and lipotoxicity (4) states after OT-CD staining. (**B**) Dynamic process of cytokinesis during cell division of HeLa cells after OT-CD staining, and the dotted line traces the cell outline. Scale bar: 10 μm.

As illustrated in Figure 6A, we utilized OT-CD to examine the distribution of LDs within cells under normal and pathological states. Figure 6A1 shows the normal distribution of LDs within Hela cells (blank group). HeLa cells were cultured in serum-free HBSS medium for 4 h to establish a starvation mode model [52, 53]. Within starvation group, we observed an increase in the density of intracellular LDs and a decrease in the brightness of LDs (Figure 6A2). This result is consistent with previous report, indicating that cells actively accumulate free fatty acids into LDs to sustain energy supply and other physiological activities during a short period of starvation. Next, to induce oxidative stress, we introduced 10 mM H_2_O_2_ into the medium, triggering the production of reactive oxygen species (ROS) in HeLa cells [54] (Figure 6A3). Compared to the blank group, the density of intracellular LDs was higher and individual LD appeared larger under oxidative stress conditions due to LDs’ ability to prevent further cell damage by merging with lipid-soluble peroxides produced by mitochondria. After incubating the cells with 4 μg ml^−1^ of oleic acid for 6 h to induce lipotoxicity [55], we observed a significant increase in LDs density and a more uniform and smaller volume of LDs (Figure 6A4). This phenomenon is attributed to the active uptake of fatty acids abundant in the environment and their storage in LDs to avoid toxicity. These findings suggest that OT-CD reliably targets intracellular LDs and exhibits almost uniform fluorescence emission, which effectively fulfills the requirement for observing the macroscopic distribution of intracellular LDs. Finally, we employed OT-CD to analyze the distribution of LDs in HeLa cells undergoing cytokinesis (Figure 6B). We observed a high density of LDs at the division site, potentially providing raw material for cell membrane production and facilitating rapid fusion between small and dense LDs upon completion of cytokinesis. This further confirms the capability of OT-CD to meet the requirements of dynamic observation.

Considering that the fluorescence peak emission position of OT-CD is sensitive to the change of solvent polarity, it may have the ability to record the change of polarity in intracellular LDs. Consequently, we recorded the fluorescence change process of LDs in Hela cells during ferroptosis continuously.

Ferroptosis is a process in which free radicals (ROS) accumulate through the Fenton reaction, leading to the conversion of unsaturated lipids to peroxidized lipids due to certain impairments (such as loss of peroxidase activity), ultimately resulting in cell death [56–58]. Because LDs function as storage centers for unsaturated lipids in cells, they may be more susceptible to ferroptosis, as indicated by the significant increase in LDs viscosity during this process [10, 59, 60]. Therefore, we suggest that when the central lipid within the LDs is oxidized, there will be an increase in overall polarity of the LDs, resulting in red-shifted OT-CD fluorescence signals.

**Figure 7.**
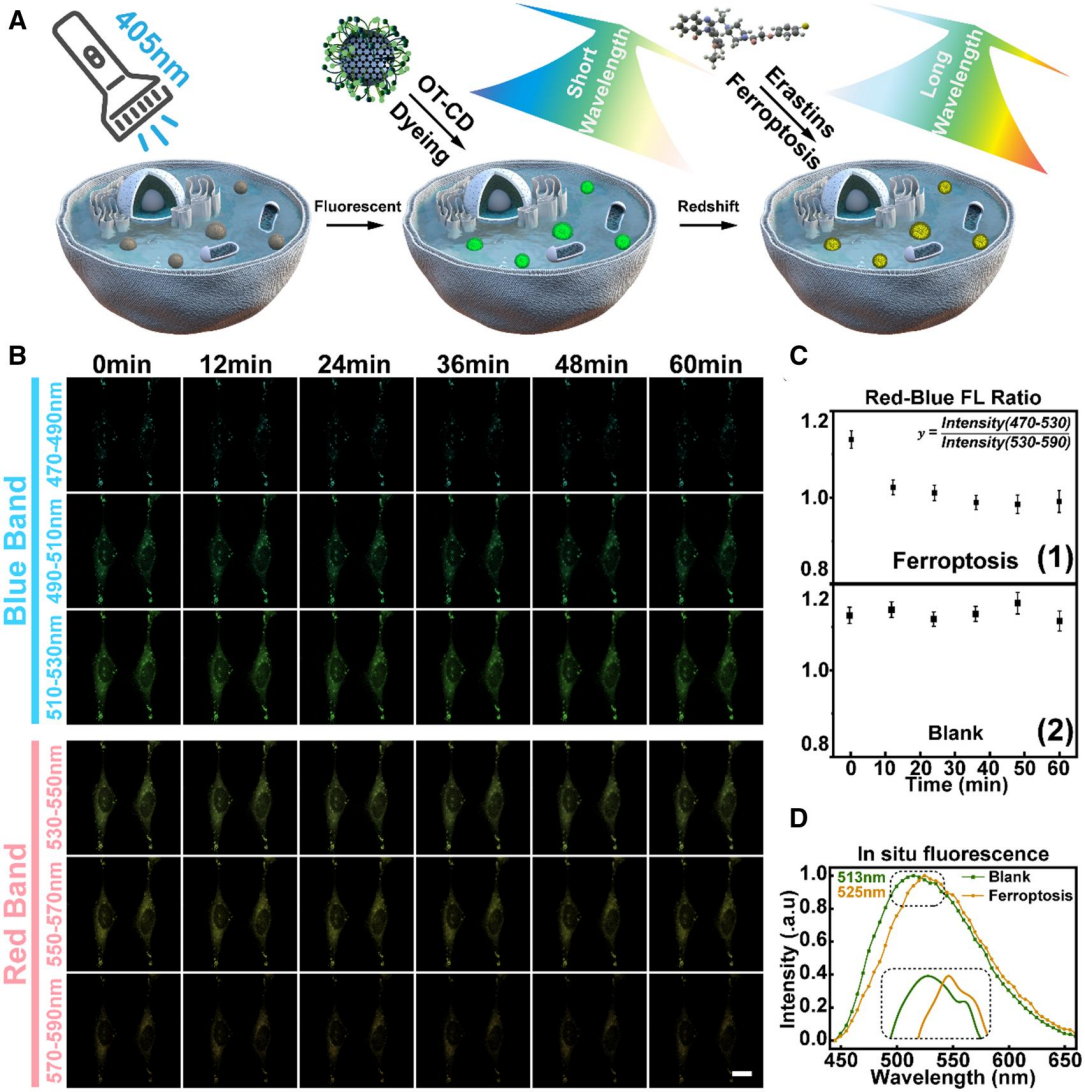
(**A**) Schematic representation of the subtle changes in fluorescence of OT-CDs in LDs during ferroptosis. (**B**) Fluorescence images at each band during ferroptosis for 1 h. (**C**) The change of fluorescence intensity ratio of blue-red band during 1 h of erastin-added group and control group. (**D**) *In situ* fluorescence spectra of LDs in the erastin-added group versus the control group after 1 h incubation (inside the dashed box is a zoomed-in view of some of the curves). Scale bar: 20 μm.

Hence, OT-CD was firstly added to Hela cells and incubated for 30 min to ensure uniform distribution, and then Erastin was added to induce the formation of ferroptosis [59–61] (Figure 7A). To mitigate the phototoxic effects of the 405 nm laser, we recorded fluorescence signals at 470–590 nm in five bands, reducing the number of exposures needed for the experiment. Variation in fluorescence intensity data collected at different bands would indicate a change in polarity of LDs. As depicted in Figure 7B, we collected average fluorescence intensity signals at each wavelength in cellular LDs every 12 min, repeating this process for the control group without Erastin addition (Supplementary Figure S9).

The fluorescence signal emitted from LDs in control cells remained stable in all wavebands (Supplementary Figure S10). However, there was a significant change in the brightness of LDs fluorescence in the cells treated with Erastin (Figure 7B). In the first four recorded images, the signal shows an increasing trend in most wavebands, with the largest relative increase observed at 530–570 nm (Supplementary Figure S11). In the last two recordings, the signal at 470–550 nm decreased significantly, while the signal at 550–590 nm remained relatively stable. The observed increase in fluorescence brightness could be due to a slight increase in LDs volume during the early ferroptosis stages. The different variation between longer and shorter wavelengths suggests an increase in LDs polarity, and to visually represent these variations, we listed the ratio of fluorescence intensity at both 470–510 nm (blue band) and 530–570 nm (red band). The Erastin-treated signal showed a decreasing trend during the first 36 min and then remained stable (Figure 7C), while the ratio of the control group fluctuated only slightly.

We then conducted *in situ* spectroscopic measurements of LDs in cells, which once again displayed at least 10 nm red-shift in OT-CD fluorescence within the LDs subsequent to ferroptosis occurrence (Figure 7D). These observations imply significant and irreversible change in polarity during ferroptosis, potentially indicating the transition process from unsaturated to peroxidized lipids.

### Zebra fish imaging

**Figure 8.**
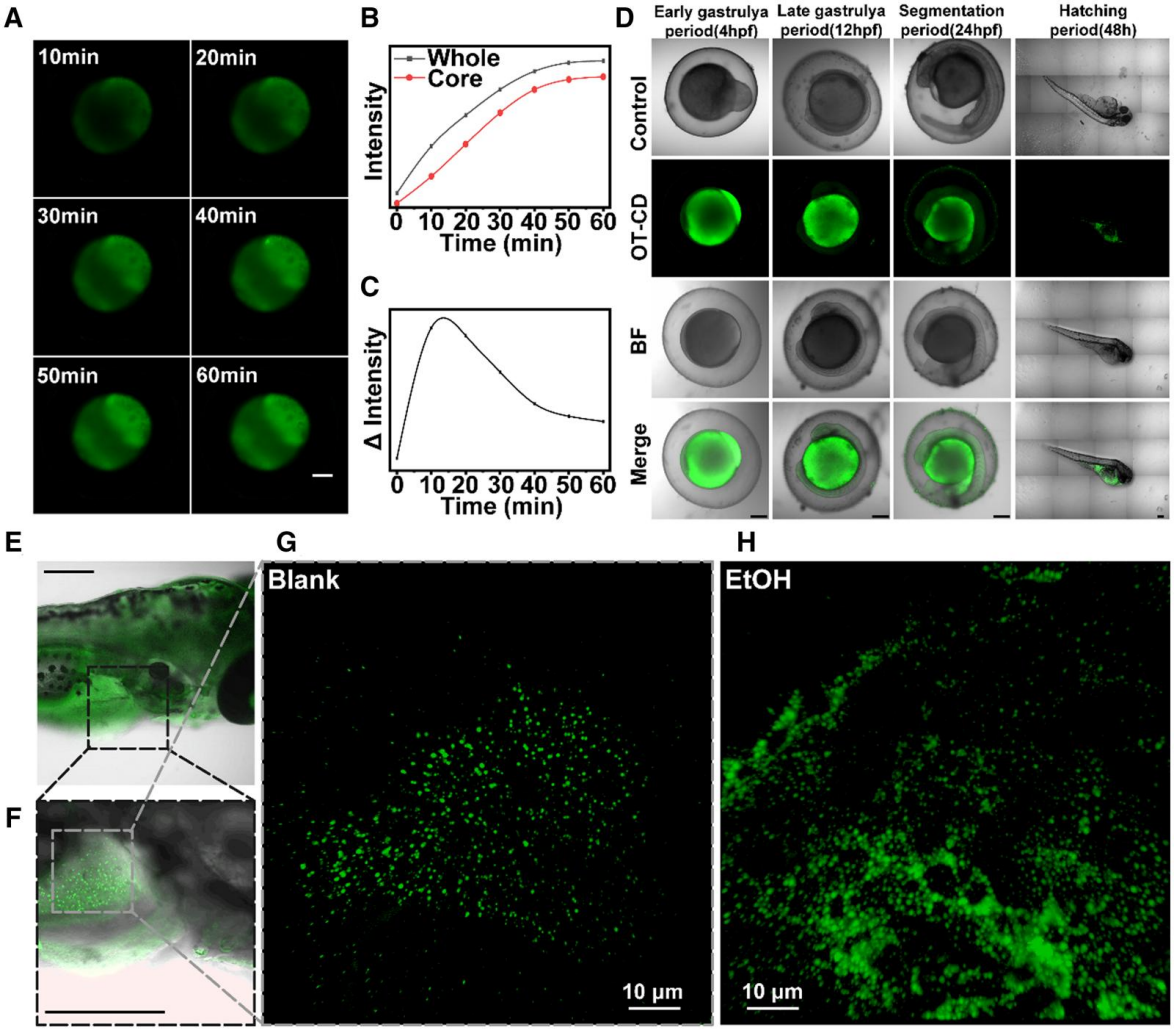
(**A**) FLSM images of zebrafish embryos at different times. (**B**) Mean fluorescence intensity over time for the whole embryo as well as the embryo center. (**C**) Mean fluorescence intensity difference between whole embryo and embryo center over time. (**D**) CLSM of zebrafish at different stage stained with OT-CDs. (**E**, **F**) CLSM images of zebrafish liver at different magnifications. (**G**) 3D fluorescence images obtained by laminar scan at the liver of normal zebrafish. (**H**) 3D fluorescence images obtained by laminar scan of liver of zebrafish with 2% ethanol-induced liver damage. Scale bar: 200 μm.

OT-CD should be well-suited for staining large volumes of biological samples with high internal lipid content structures due to its excellent amphiphilicity and high lipid–water partition coefficient. To validate its fast-imaging capabilities, we utilized the zebrafish model, which is optically transparent and allows for easy observation of internal organs, tissues and subcellular organelles through fluorescence imaging despite its larger size relative to cellular samples. Since zebrafish embryos are much larger than cells, we determined the appropriate OT-CD staining concentration of 20 μg ml^−1^ by pre-experimentation. As depicted in Figure 8A, we observed clear, bright fluorescent signals in the core of zebrafish embryos within 10 min of OT-CD addition, with very distinct presentation visible at 30 min, a time comparable to conventional cell imaging. We quantified the average fluorescence intensity in the images across both the whole embryo and its center (Figure 8B). The synchronous increase in fluorescence intensity at the center and throughout the entire embryo suggests that after entering the surface of the embryo, OT-CDs in water immediately diffuse toward the center owing to their excellent amphiphilicity (Figure 8C). Then we listed the variation in the difference between the average fluorescence intensity of the embryo as a whole and at its center, which reached maximum value within 10–15 min and remained stable thereafter, indicating that OT-CD can passively penetrate biological samples of approximately 0.4 mm thickness within 20 min. In addition, as shown in Figure 8D, zebrafish embryos were labeled and yolk sac morphology was recorded at different developmental periods using OT-CD and did not cause visible developmental malformations in zebrafish larvae even two days after staining.

Next, we observed the content of LDs in the liver of zebrafish with ethanol-induced liver injury. Figure 7E and F shows stepwise magnification of macroscopic images of zebrafish, indicating the location of our final observed liver. Figure 7G and H shows FLSM images of zebrafish in the blank and ethanol-induced liver injury groups. Comparison of laminar scan images at specific areas confirmed that treatment with 2% ethanol resulted in more LDs at the liver location of zebrafish. These results indicate that OT-CDs can rapidly diffuse in both aqueous and oily media, ultimately emitting bright fluorescence in tissues with high fat content.

## Conclusions

In summary, through a comparative screening of CDs synthesized using the solvothermal method under various conditions, we successfully obtained a CD that is specific to LDs, which can be synthesized under relative milder condition of 180°C and exhibits excellent optical properties. This OT-CD is characterized with abundant surface fluorophores and well-developed carbonized cores, and thus exhibits remarkable amphiphilicity, solvatochromic, high PLQY, excellent photostability and good biocompatibility. Benefiting from that, OT-CD facilitates wash-free imaging and enables long-term stable observation. By utilizing OT-CD as the imaging probe, we successfully documented the distribution and morphology of LDs in both physiological and pathological cellular states. Additionally, we observed an increase in the polarity of LDs during ferroptosis. Lastly, we documented the accumulation of lipids at the ethanol-induced liver injury in zebrafishs. We believe that the findings of this study not only inspire the development of synthetic routes for CDs as fluorescent probes but also broaden the scope of their applications. Moreover, they provide a valuable visible tool for studying related diseases process effectively.

## Supplementary Material

rbad109_Supplementary_Data
