# Supplementary material for: Mild synthesis of ultra-bright carbon dots with solvatochromism for rapid lipid droplet monitoring in varied physiological processes
Source: Regen Biomater. 2024 Jan 16;11:rbad109. doi: 10.1093/rb/rbad109 (PMC10884737; doi:10.1093/rb/rbad109)
Supplement: rbad109_Supplementary_Data [file rbad109_supplementary_data.zip › Final Supporting_Information.docx]

Supporting Information

**Mild Synthesis of Ultra-Bright Carbon Dots with Solvatochromism for Rapid Lipid Droplet Monitoring in Varied Physiological Process**

Borui Su^1^, Dong Gao^1^, Nini Xin^1^, Kai Wu^1^, Mei Yang^1^, Shichao Jiang^1^, Yusheng Zhang^1^, Jie Ding^1^, Chengheng Wu^1,2^, Jing Sun^1^, Dan Wei^1^, Hongsong Fan^1,^*, Zhenzhen Guo^3,^*

** Corresponding authors: Hongsong Fan (hsfan@scu.edu.cn); Zhenzhen Guo (zhenzhenguo0225@163.com)*

*1. National Engineering Research Center for Biomaterials, College of Biomedical Engineering, Sichuan University, Chengdu 610064, Sichuan, China.*

*2. Institute of Regulatory Science for Medical Devices, Sichuan University, Chengdu 610064, Sichuan, China.*

*3. Department of Gastroenterology, Sichuan Provincial People’s Hospital, University of Electronic Science and Technology of China, Chengdu, China*

**Supplementary Videos**

**Video S1** shows Blood flow and heartbeat of zebrafish larvae shown by rapid dynamic imaging.


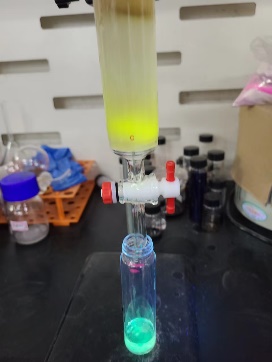


**Figure. S1** Fluorescent appearance of OT-CDs in silica gel column and DCM irradiated with 365 nm UV.


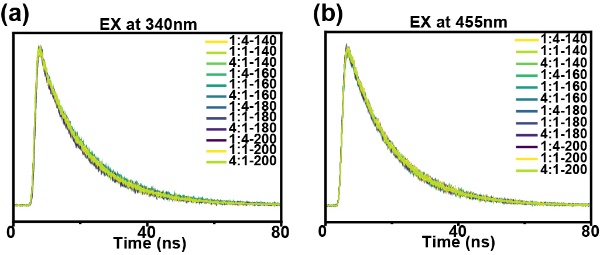


**Figure. S2** Fluorescence lifetime curves of OT-CDs obtained under different synthesis conditions under 340 (a) and 455 nm (b) laser excitation.





**Figure. S3** Excitation-emission matrix of the new fluorescent component emerging from OT-CD obtained with 4:1/180°C as synthesis condition and then heated at 180°C for 8h.


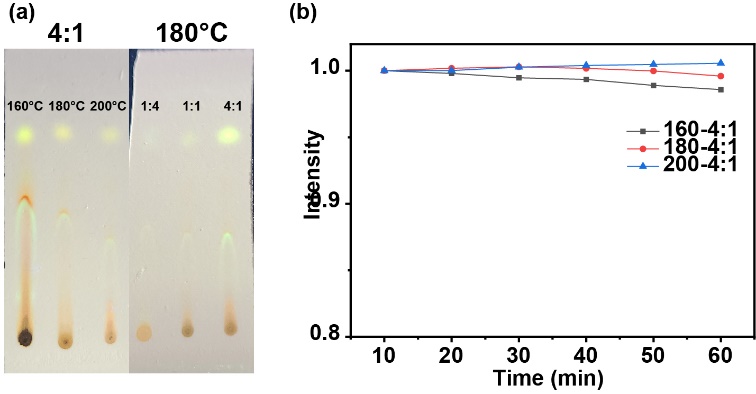


**Figure. S4** (a) Yield of OT-CDs under different synthesis conditions. (b) Characterization of the photostability of OT-CDs obtained at three synthesis temperatures.


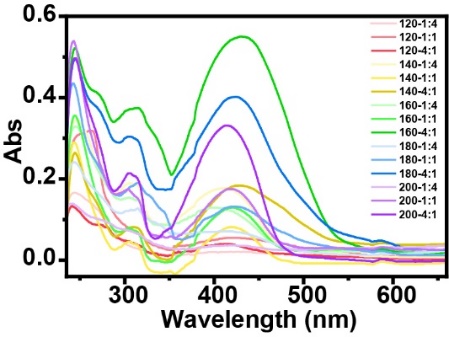


**Figure. S5** UV-Vis absorption spectra of OT-CDs obtained under different conditions.


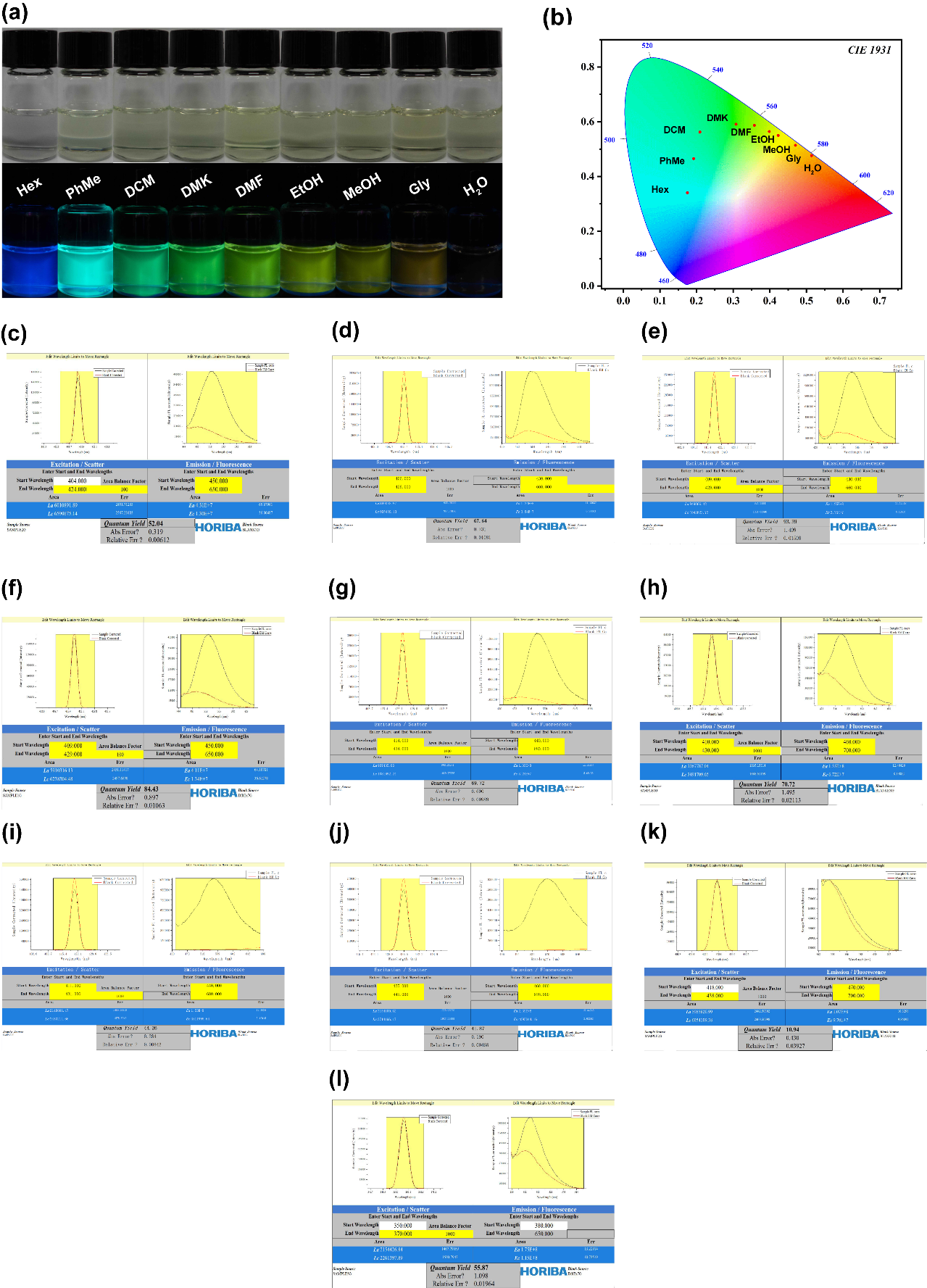


**Figure. S6** Fluorescence performance of OT-CD in different solvents. (a) Appearance of OT-CD in different solvents under sunlight and 365 nm UV irradiation. (b) CIE1931 coordinates of OT-CD fluorescence in different solvents. The absolute PLQY of OT-CD in (c) Hex, (d) PhMe, (e) DCM, (f) DMK, (g) DMF, (h)EtOH, (i) MeOH, (j) Gly, (k) H_2_O and (l) quinine sulphate.


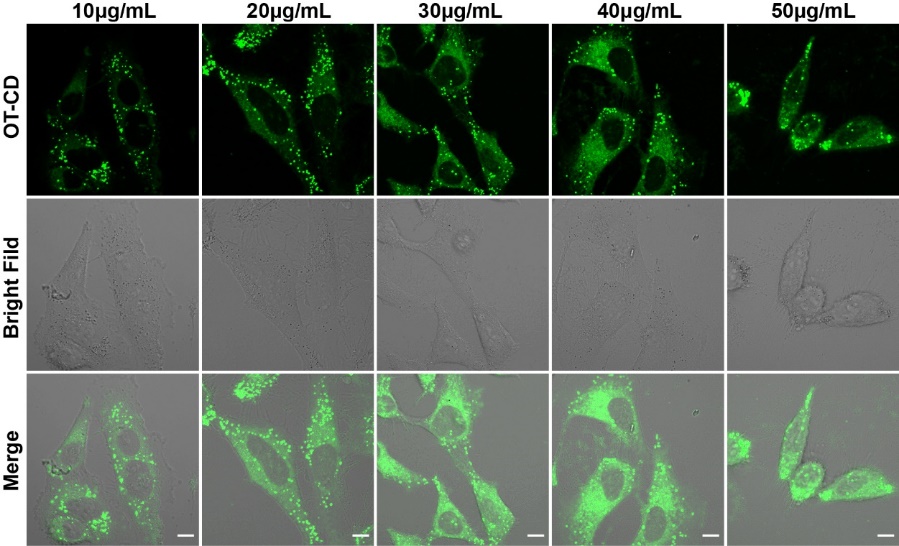


**Figure. S 7** Fluorescence images of Hela cells with different concentrations of OT-CD.


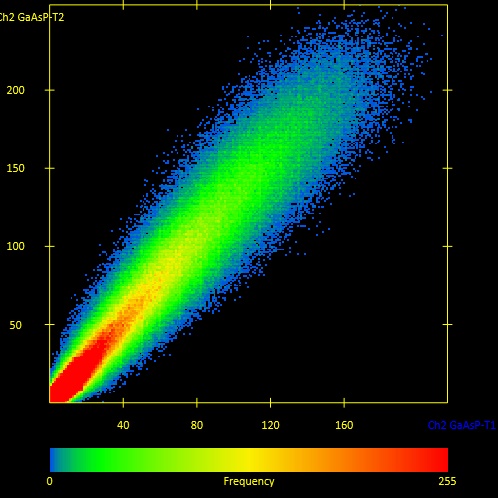


**Figure. S8** Overlap assessment of co-localization of OT-CD and BODIPY.


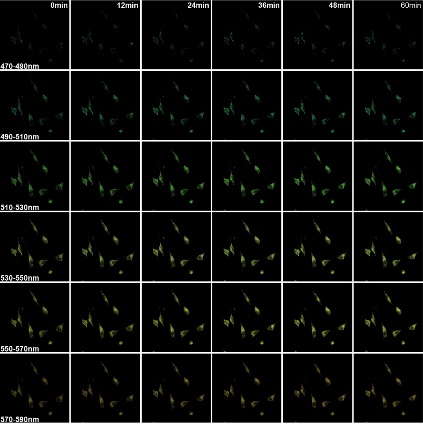


**Figure. S9** Fluorescence images of Hela cells in blank group without Erastin in each band within 1h.


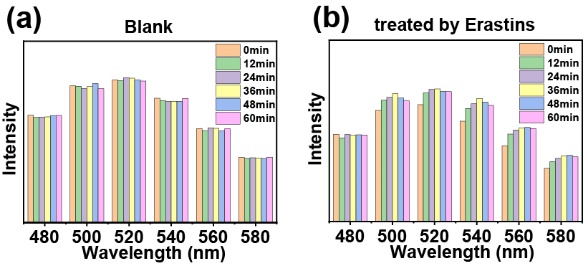


**Figure. S10** The course of fluorescence intensity of each waveband over time in the control group (a) and the experimental group (b) in the ferroptosis experiment over a 1-h period.

**Table S1** Photophysical properties of OT-CDs in various solvents

| Solvent | ET(30) | λ_ex_  (nm) | λ_em_  (nm) | Stokes shift  (nm) | QY | FWHM  (nm) | Lifetime  (ns) |
| --- | --- | --- | --- | --- | --- | --- | --- |
| Hex | 30 | 402 | 489 | 87 | 52.04 | 74 | 6 |
| PhMe | 33.9 | 416 | 495 | 79 | 67.64 | 68 | 18.1 |
| DCM | 40.7 | 416 | 507 | 91 | 93.39 | 82 | 21.3 |
| DMK | 42.2 | 419 | 525 | 106 | 84.43 | 87 | 19.3 |
| DMF | 43.2 | 424 | 537 | 113 | 69.72 | 91 | 21.8 |
| EtOH | 51.9 | 420 | 548 | 128 | 70.72 | 94 | 17.3 |
| MeOH | 55.4 | 421 | 554 | 133 | 44.26 | 99 | 11.3 |
| Gly | 57 | 435 | 570 | 135 | 41.87 | 102 | 10.5 |
| H_2_O | 63.1 | 428 | 585 | 157 | 10.94 | 114 | 7.1 |
